# Effect of drill quality on biological damage in bone drilling

**DOI:** 10.1038/s41598-023-33381-y

**Published:** 2023-04-17

**Authors:** Khurshid Alam, Sayyad Zahid Qamar, Muhammad Iqbal, Sujan Piya, Mahmood Al-Kindi, Asim Qureshi, Ahmed Al-Ghaithi, Badar Al-Sumri, Vadim V. Silberschmidt

**Affiliations:** 1 Department of Mechanical and Industrial Engineering, College of Engineering, Sultan Qaboos University, P.O. Box 33, Al-Khoud, 123 Sultanate of Oman; 2 Creative Engineering & Management Services, Saddar Road, Peshawar, Pakistan; 3 Department of Industrial Engineering and Engineering Management, College of Engineering, University of Sharjah, Sharjah, UAE; 4 Department of Pathology, Sultan Qaboos University, Al-Khoud, 123 Sultanate of Oman; 5 Department of Surgery, Sultan Qaboos University Hospital, Al-Khoud, 123 Sultanate of Oman; 6 Histopathology Laboratory, Sultan Qaboos University Hospital, Al-Khoud, 123 Sultanate of Oman; 7 School of Mechanical, Electrical and Manufacturing Engineering, Loughborough University, Loughborough, UK

**Keywords:** Biomedical engineering, Health care, Engineering

## Abstract

Bone drilling is a universal procedure in orthopaedics for fracture fixation, installing implants, or reconstructive surgery. Surgical drills are subjected to wear caused by their repeated use, thermal fatigue, irrigation with saline solution, and sterilization process. Wear of the cutting edges of a drill bit (worn drill) is detrimental for bone tissues and can seriously affect its performance. The aim of this study is to move closer to minimally invasive surgical procedures in bones by investigating the effect of wear of surgical drill bits on their performance. The surface quality of the drill was found to influence the bone temperature, the axial force, the torque and the extent of biological damage around the drilling region. Worn drill produced heat above the threshold level related to thermal necrosis at a depth equal to the wall thickness of an adult human bone. Statistical analysis showed that a sharp drill bit, in combination with a medium drilling speed and drilling at shallow depth, was favourable for safe drilling in bone. This study also suggests the further research on establishing a relationship between surface integrity of a surgical drill bit and irreversible damage that it can induce in delicate tissues of bone using different drill sizes as well as drilling parameters and conditions.

## Introduction

Drilling of bone with a hard metallic drill is a common surgical procedure used in various contexts in orthopaedics, neurosurgery, and dentistry. During the drilling process, a cylindrical hole is produced to accommodate screws or other fasteners for rigid fixation. Mechanical fasteners holding fragments of bone together can resist the axial and shear forces and support the skeleton during locomotion. Despite technological advancement in surgical procedures and development of robot-assisted systems for cutting bones, drilling with a hand-held surgical drill is still a preferred choice in clinics. As a result, performance of skeletal fixation mainly depends on the skill of the surgeon, type of bone, as well as drilling parameters and conditions.

High magnitudes of drilling force and torque as well as a temperature rise above a normal physiological levels are the inevitable outcomes of drilling in bone^1–10^. One of the main reasons for overheating of bone during the drilling process is low thermal conductivity of bone tissue. Excessive heat and large drilling forces can cause osteonecrosis of the tissue and breakage of drill during the procedure^5,11,12^. Large drilling forces were reported to cause microcracks in the immediate vicinity of the drilled holes and damage of a delicate structure of bone and soft tissues near the drilling region^1^. Heat generation in bone was shown to depend strongly on drill size, drilling speed, feed rate, and axial thrust force^13–15^, while the geometrical parameters of a drill bit such as point, rake and helix angles affected the bone temperature, thrust force and torque^16,17^. Still, the research published on the topic has no consensus on optimum drilling parameters for safe and efficient drilling in bone.

It was long known that a temperature rise in bone above the thermal threshold level can cause its necrosis (death). Necrosis of bone is the cell death due to an irreversible external injury that may result in the collapse of bone architecture. In histologic images it is manifested by a series of empty osteocytic lacunae, which can seriously affect the osteogenic potential of bone surrounding the implant. Depending on the total time of exposure of the bone tissue to heat, a range of reported values of temperature (47 °C to 70 °C) could produce irreversible thermal damage in bone^5,13^. Mechanical factors such as shear stress, hydrostatic pressure and structural deformation can also induce physiological changes in bone cells^18,19^.

Bone drilling is usually performed for a short interval of time and, currently, there is no study investigating the effect of drill load on post-operative remodeling of bone surrounding the implant. Although temperature was found to be the most important indicator for inducing thermal necrosis in bone, the drilling force and torque were also reported as main contributors affecting postoperative outcomes. In a recent study^20^, cell damage in bone during drilling was found to be a combined result of drilling force, temperature and torque. Thermal thresholds for necrosis in bone drilling are presented in some review articles^5,13,21,22^. To study the effect of drilling, a traditional histological study could be complemented with micro-mechanical analysis to assess the extent of damage in bone during drilling^23^. A lower drilling speed with higher pressure was found suitable for better bone regeneration^24^. The use of a conical drill was reported to produce lower temperature and, consequently, improved the healing of bone tissue compared to a cylindrical drill^25^.

Recent research on bone drilling has been mainly focused on exploring novel drilling techniques for minimum invasive surgical incision. One such technique, in which microvibrations are imposed on the drill along the feed direction, is known as vibrational drilling (VD) or ultrasonically-assisted drilling (UAD). Contrary to this novel drilling technique, conventional drilling (CD) is a traditional method, used for bone cutting, with the drill rotating only about its fixed axis. The new technique was successfully used in several research studies on bone drilling^26–30^. It has demonstrated a lower drilling force, minimum twisting resistance and lower temperature in bone compared to CD. Drilling in bone assisted by ultrasonic vibrations showed reduction in delamination at the entrance point around the hole^31^. Some studies found a lower cutting force and an elevated temperature when vibrational plane cutting and orthogonal elliptical vibration-assisted cutting were used in bones^32,33^. Some recent studies evaluated the performance of UAD with regard to biological damage induced in the bone^34^. Drilling assisted by vibrations imposed on the drill was also found helpful for maintaining structural integrity of the bone^35^. In these studies, the response of the bone cells to UAD near the cutting region was studied with a limited number of drilling parameters and conditions. Unfortunately, the published studies investigated the effect of ultrasonic vibrations on the outcome of the drilling process without considering such important conditions such as drill wear and the penetration depth of the drill into the bone tissue.

As well known, a repeated use of surgical drills causes wear of their cutting edges. Blunt edges of a drill bit significantly reduce its cutting ability and accuracy, and increase the risk of its failure during the surgical operation. Some known negative outcomes of a worn drill in bone are the generation of excessive heat^36^, large drilling forces and twisting resistance^37^. Bone temperature was found to have a direct relationship with the number of times the drills was used^36^. In addition to thermal and mechanical damage in bone, the cutting edges of a worn drill can be fractured and break during a surgical incision^38,39^.

Drilling with worn cutting edges of the drill can cause structural and thermal damage to the cut material. The surgeons drill the bone without concerns about the negative outcomes of drill condition. In most bone-drilling procedures, the drill has to penetrate the full thickness of the cortex. Drilling deep into the cortical bone can induce damage invisible to the operator. Although the link between the drill wear, force, torque and temperature in a bone-drilling process is well established, to the authors knowledge, no study addressed the effect of drill wear on cell viability in bone. Also the relationship between the drilling parameters, drill wear, depth of drilling and associated biological damage is not yet established. Considering the discussed challenges in bone drilling, the primary goal of this study is to provide scientific information to clinicians about the extent of damage that a drill with different levels of roughness or wear can induce in bone at different depths of drilling.

## Materials and methods

### Bone specimens

Femoral and tibia bones of a 3-years-old freshly slaughtered cow were used in drilling experiments. No approval was required since all experiments were conducted on dead bone. Both types of bones, with hollow cylindrical structures and compact sections in the middle diaphysis, were used in experiments. The average cortical thickness of bones was 9–10 mm. Hollow cylindrical pieces of 4 to 5 cm length were cut from the main shafts of long bones for clamping them in fixture for drilling operations. The focus was on the Cortical bone since it is much denser, stronger and stiffer than cancellous bone. The average mineral density of the bone was measured with Dual-energy X-ray absorptiometry (DXA) and was found to be 1.9 g cm^−3^. Two types of specimens were used in this study. One type was used for drilling experiments, while another small drilled pieces for histology examination. A total of eight bone specimens, each accommodating between fifteen and twenty holes (depending on the size of the drill bit), were cut from main femoral and tibia shafts. Samples for drilling were wrapped in plastic bags and stored in a refrigerator at − 10 °C for about an hour. Frozen samples were allowed to attain the room temperature just before using them in drilling experiments. Drilling was only performed in specimens that were apparently compact and free of visible osteoporosis.

### Drilling equipment and procedure

**Figure 1:**
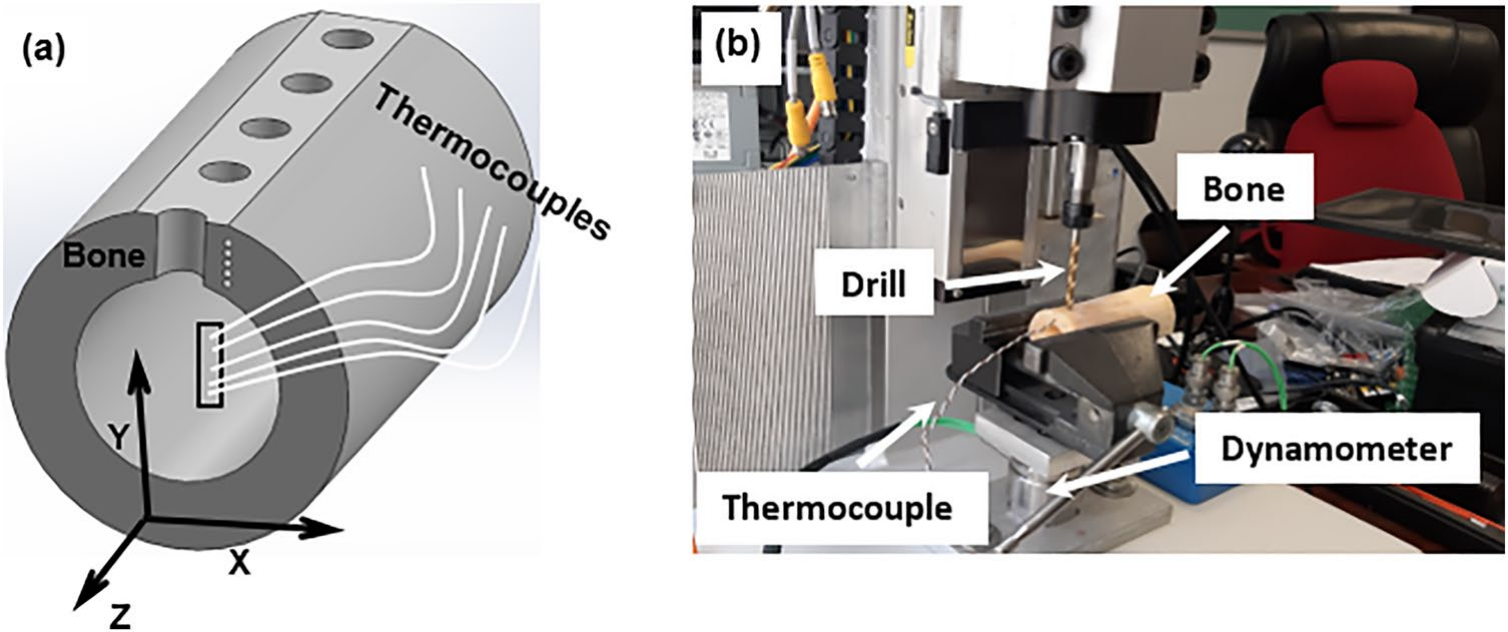
(**a**) Schematic of bone specimen with thermocouples placed through cortical thickness of bone (https://www.solidworks.com). (X—radial direction, Y—drilling direction, Z—longitudinal axis of long bone), (**b**) experimental setup for bone drilling.

**Figure 2:**
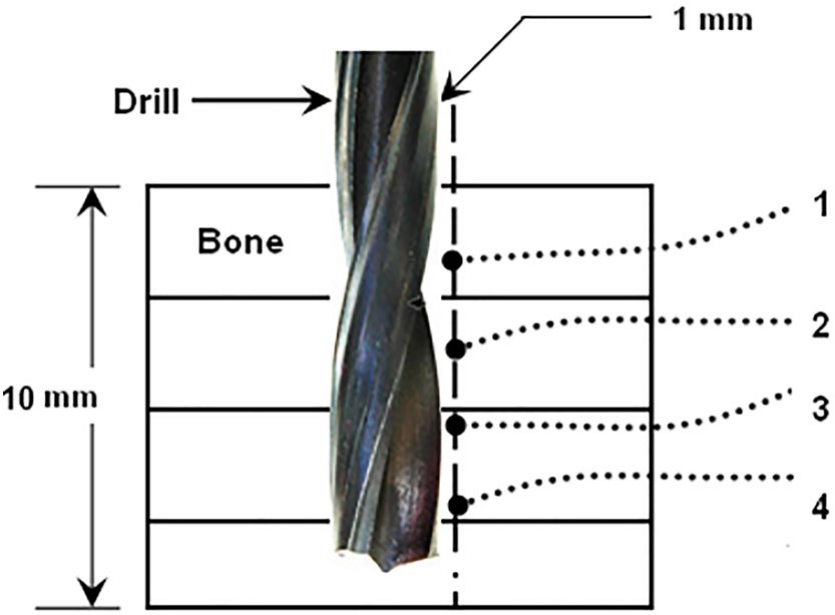
Schematic of thermocouple locations along the drilling depth.

A custom-made drilling setup with a feedback control system for force, torque and temperature was used in drilling tests (Fig. 1b). A CNC 400 W DC brushless spindle motor with a maximum driver power of 600 W and a driver running speed of 12,000 rpm was used to drive the drill bit. The levels of drilling force and torque were measured with a two-component dynamometer (type 9345B, Kistler, Switzerland) with a maximum force and torque capacity of ± 10 kN and ± 25 N∙m, respectively. Drilling tests were performed in the presence of gentle spray of saline water over the drilling area. The saline solution was used to keep the drilling region cool as well as to assist the evacuation of bone chips without clogging in the drilling track. The cooling is expected to have little influence on the drilling force and torque. The feed rate was fixed at 40 mm/min in all experiments. All drilling tests were conducted at room temperature of about 25 °C. Type K thermocouples (temperature range − 40 °C to 500 °C) were used to measure temperature during the drilling operation. Small holes of 1.5 mm diameter were drilled on one side of the cross-section of specimens for placing the bead of the thermocouple (see Fig. 1a). The 3D model of bone shown in the Figure was created using Solidworks software (Solidworks 2018, Dassault Systemes). These holes were produced using an extremely slow speed of 400 rpm in the presence of spray of water to keep the drilling region cool. Thermocouples were placed at depths of 4 mm, 5 mm, 6 mm, and 7 mm from the top surface of the bone specimen for each drilling test; they were placed at a distance of 1 mm from the surface of the hole (Fig. 2). It took 15 to 20 min for the thermocouple holes to be drilled in the specimen since a specified depth for each hole was to be controlled. The waiting time between the two drilling stages was approximately 5 to 10 min taken by setting the specimen for drilling.

### Drill wear measurement

**Figure 3:**
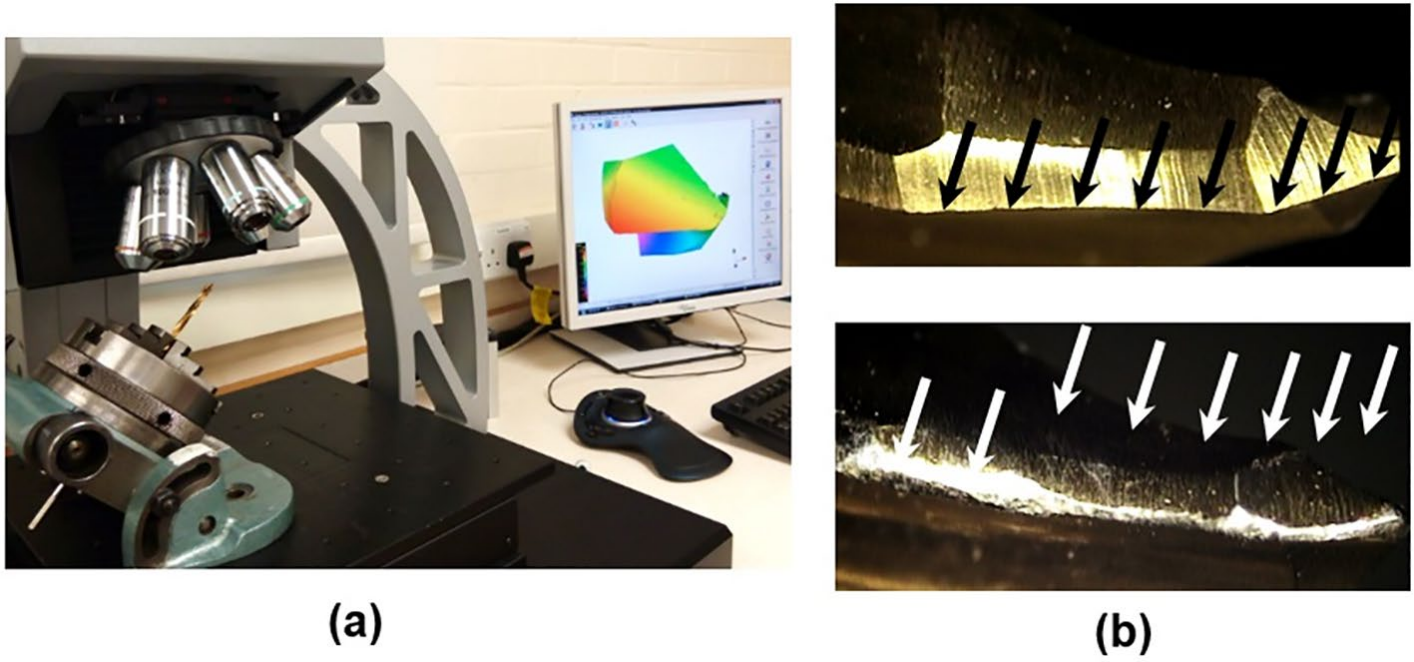
(**a**) Measurement of roughness of drills; (**b**) cutting edges (showed with arrows) for surface- roughness measurements.

Drilling tests were carried out using standard two-flute 4.8 mm surgical drills (Orthofix, Italy). There is no established technique in orthopedic surgical drilling to assess the surface quality of drills. The discard of a surgical drill is either based on visual appearance of its cutting edges on sensation of drilling forces experienced by the surgeon. In this study, roughness of cutting lips of the drills was measured using an OGP Flash 200 Optical measuring microscope and Alicona Infinite Focus microscope (see Fig. 3). Several drill bits, including sharp (new) ones, of the same size were donated by an orthopedic department of a local hospital, and the surface roughness was measured for each drill bit. The obtained scan data were used to generate a 3D map of the surface of the cutting edges, chisel edges and the flank. The approximate number of holes produced by each drill before the measurements presented in Table 1 was based on the opinion of experts in orthopedic surgical procedures. The average values of surface roughness presented in Table 1 are rounded. The roughness profiles from the 3D scan data were obtained using Alicona IFM 3.5.01 software. For this purpose, the roughness profile was obtained along the cutting edges (cutting lips) and chisel edges of the drills. Five drill bits with known numbers of drilled holes and average values of roughness were used in the drilling experiments (Table 1).

**Table 1:** Approximate values of roughness of surgical drills.

Drill category	Approximate number of holes	Average roughness, *Ra* (µm)
Sharp drill	0	1
Worn drill—1	50	2
Worn drill—2	100	3
Worn drill—3	150	4
Worn drill—4	200	5

### Specimens for histological examination

Tissue processing is the steps required to take the tissue from the fixation step to the state where it is ready for section cutting on the microtome, which requires passing through different infiltration solutions ending with a suitable histological wax and can be embedded and blocked in wax blocks. Specimens with drilled holes were fixed in a 10% formaldehyde solution for 10 h, followed by decalcification in a solution of 40 ml 65vol% nitric acid, 20 ml 10vol% formaldehyde and 340 ml distilled water for 2 days. This was performed to visualize osteocytes, which is impossible without dissolving bone minerals of the matrix. As well known, decalcification only dissolve minerals without affecting histology of the bone. Decalcification should was done before the start of the processing steps. Slices were cut at the same levels from the top surface of the specimens where the thermocouples were placed. Viable and dead osteocytes in the microstructure of the slices were visualized using a BX53 Olympus microscope.

### Statistical analysis

This section presents a detailed statistical analysis implemented to identify favorable drilling parameters with regard to safety. For statistical calculations, an area enclosed by a ring with width of only 200 µm from the edge of the hole was chosen. Each drilling test was carried out three times to ensure repeatability in the test data. Three experimental parameters and their five levels used in the experiments are provided in Table 2. Mini-tab (version 19) software used for statistical analysis. Note that the experimental parameters and their levels were decided based on the past literatures and opinion of experts from relevant fields. It is believed that these parameters will have a significant impact on the biological structure of bone during drilling process. Two data sets were considered for experiments. For one data set, the drilling depth was kept constant at 5 mm, while the values of drill speed and drill roughness were varied. In this case, considering a full factorial design, 25 experimental setups were generated. Similarly, for the second data set, the drill speed remained fixed at 2000 rpm, while the levels of drilling depth and drill roughness were changed. Here, further 25 experimental setups were generated. Obviously, out of these 50 experimental setups, five were the same. Therefore, omitting similar setups, in total, 45 experiments were conducted for the statistical analysis. The effect of these variations on multiple response variables such as drilling force, torque, bone temperature and cell loss was studied.

**Table 2:** Levels of parameters in bone-drilling experiments.

Parameter (units)	Level 1	Level 2	Level 3	Level 4	Level 5
Drill speed (rpm)	1000	1500	2000	2500	3000
Drill roughness (µm)	1	2	3	4	5
Depth of drilling (mm)	3	4	5	6	7

A grey relational analysis (GRA) method was used to check the effect of drilling parameters on the multiple response variables. GRA is widely used to optimize the multi-process parameters for multiple response variables^40^. By using it, multiple response variables can be converted into a single grey relational grade (GRG), irrespective of whether the response needs to be maximized or minimized^41^. Following steps are followed to convert multiple response variables into GRG:

*Step 1 Normalizing the response variables between* (0, 1) In this research, all four response variables should be minimized to improve the drilling process. The responses were normalized to a linear scale from 0 to 1, with the maximum value at 0 and the minimum at 1, using the following equation:

1 $$X_{ij} = \frac{{Y_{ij} - \min (Y_{ij} )}}{{\max (Y_{ij} ) - \min (Y_{ij} )}},$$

where *i* is the response variable (1, 2,…, *n*); *j* is the experiment number (1, 2,…, *m*); *Y*_*ij*_ are the observed data for response *i* at experiment *j*; *X*_*ij*_ is the normalized value of* Y*_*ij*_.

*Step 2 Calculate the grey relational coefficient* The grey relational coefficient helps to express the relationship between the normalized experimental results with the ideal result. It is calculated as

2 $$G_{i,j} = \frac{{\mathop {\min }\limits_{i} \mathop {\min }\limits_{j} \left| {X_{i}^{o} - X_{ij} } \right| + \alpha \mathop {\max }\limits_{i} \mathop {\max }\limits_{j} \left| {X_{i}^{o} - X_{ij} } \right|}}{{\left| {X_{i}^{o} - X_{ij} } \right| + \alpha \mathop {\max }\limits_{i} \mathop {\max }\limits_{j} \left| {X_{i}^{o} - X_{ij} } \right|}},$$

where $X_{i}^{o}$ is the ideal normalized value for the *i*th response variable and *α* is the distinguishing coefficient, the value of which varies in the range of (0, 1). *α* was chosen to be 0.5 to give equal preference to the maximum and minimum absolute deviations^42^.

*Step 3 Calculate the grey relational grade* The grey relational grade (*G*_*j*_) is the average of *G*_*ij*_ values for experiment *j*:

3 $${G}_{j}=\frac{1}{n}\sum_{i=1}^{n}{G}_{ij}.$$

## Results

### Temperature, force and torque measurement

**Figure 4:**
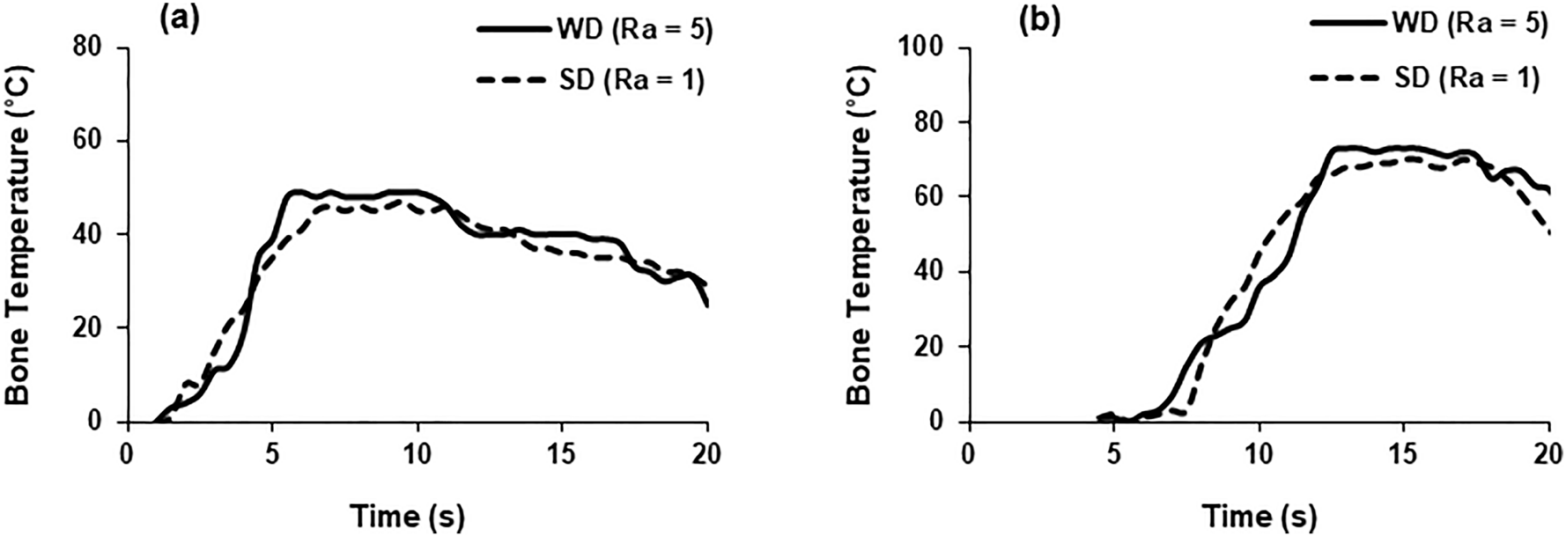
Temperature evolution in bone at depth of 3 mm (**a**) and 7 mm (**b**) (drill speed—2000 rpm, WD—worn drill, SD—sharp drill).

**Figure 5:**
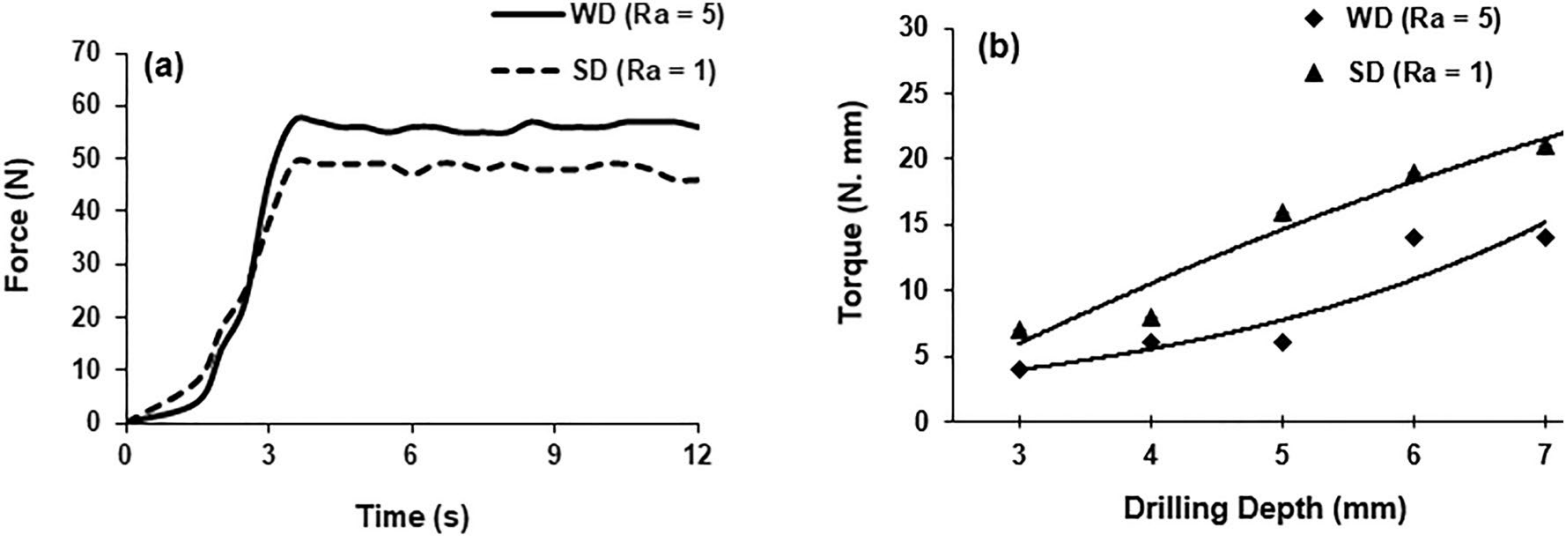
(**a**) Force evolution in bone drilling, (**b**) torque experienced by drill at different depths of drilling (drill speed—2000 rpm, WD—worn drill, SD—sharp drill).

**Figure 6:**
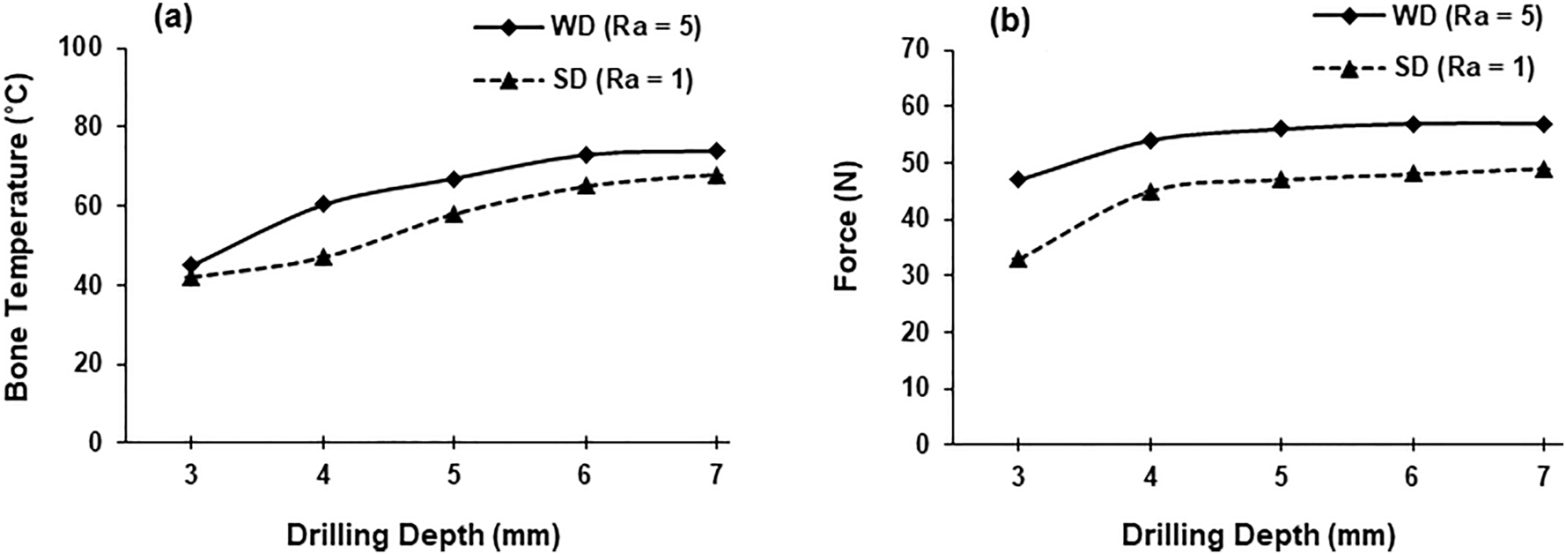
Temperature (**a**) and force (**b**) measurements at various depths of drilling (drill speed—2000 rpm, WD—worn drill, SD—sharp drill).

**Figure 7:**
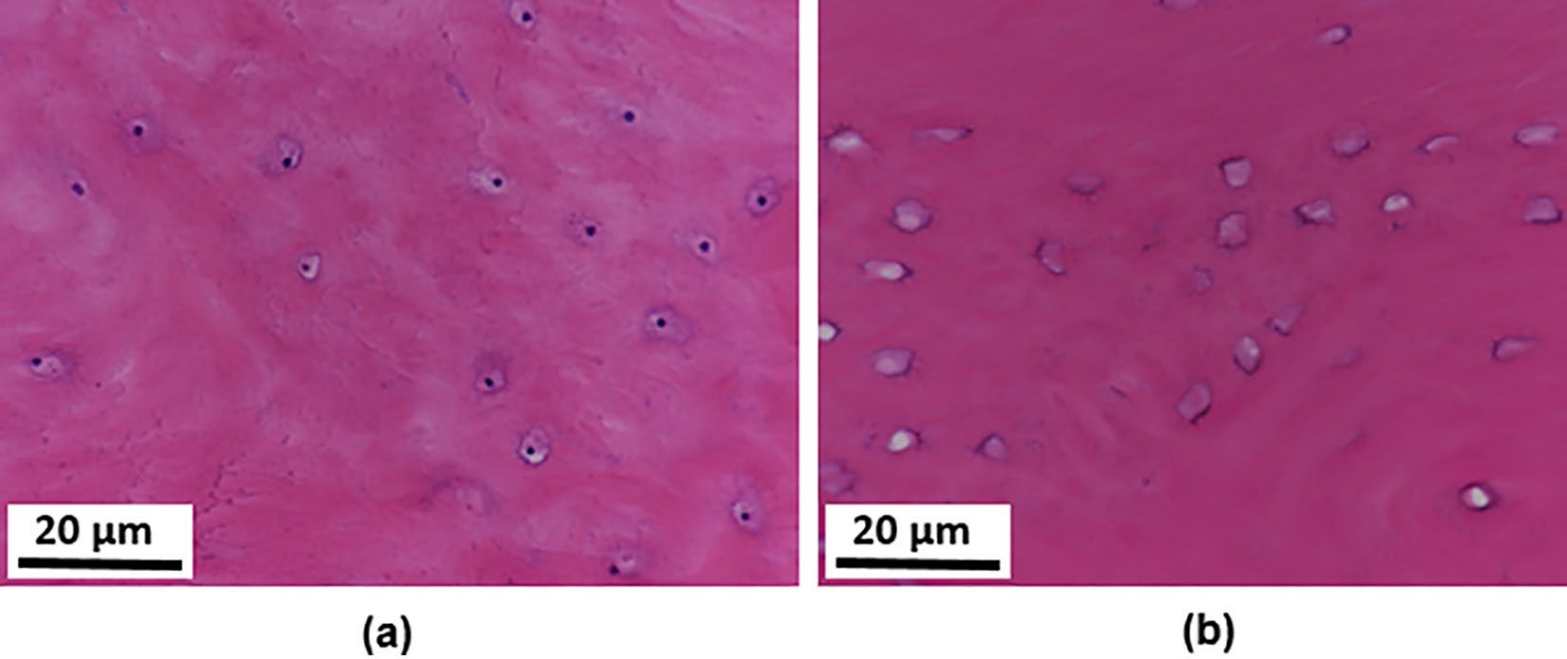
Histology plots: (**a**) lacunae with osteocytes (black dots); (**b**) empty lacunae. The image was taken at 20× zoom.

**Figure 8:**
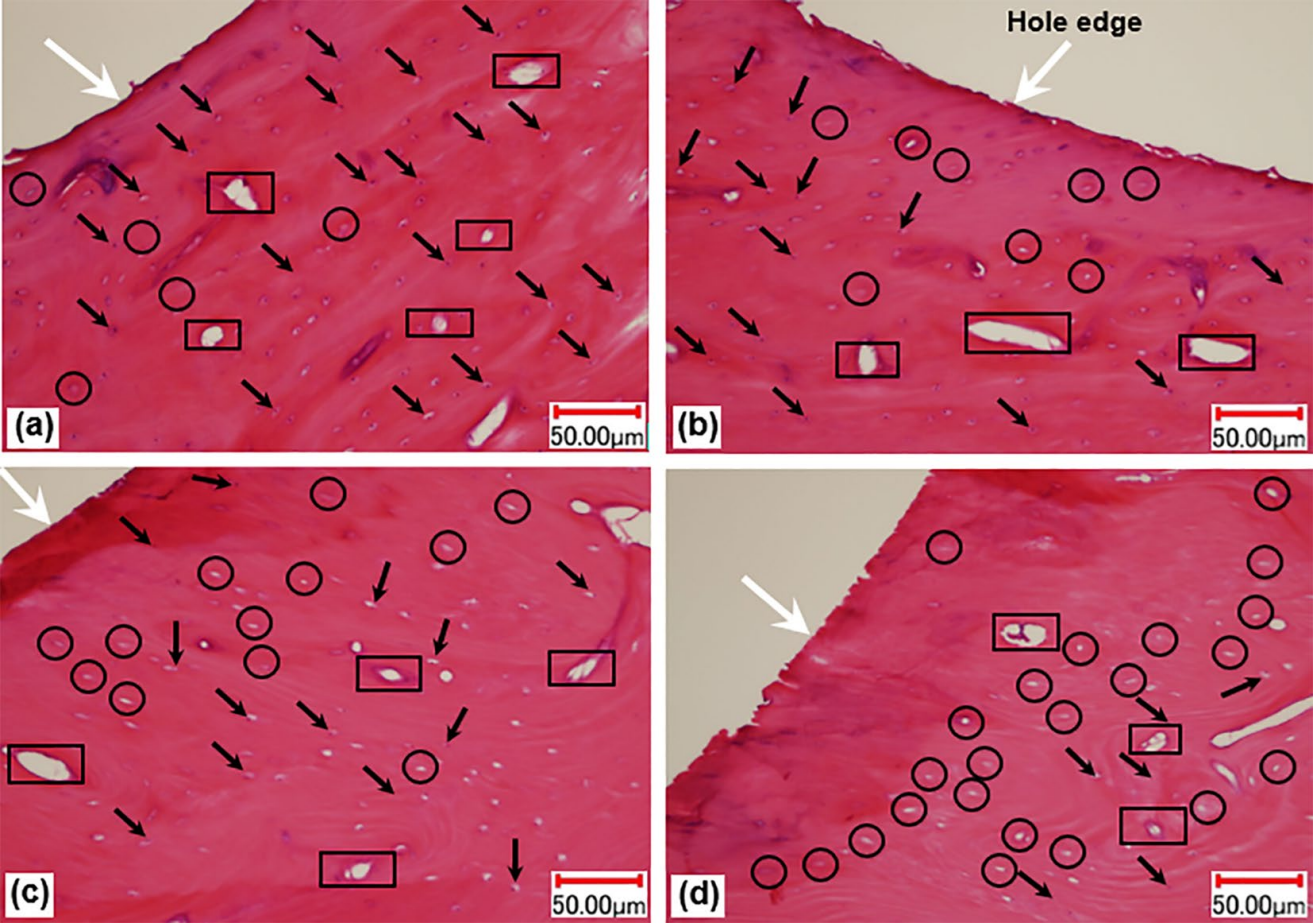
Histology plots: (**a,b**) 3 mm depth; (**c,d**) 7 mm depth ((**a,c**) sharp drill; (**b,d**) worn drill). The image was taken at 10× zoom.

**Figure 9:**
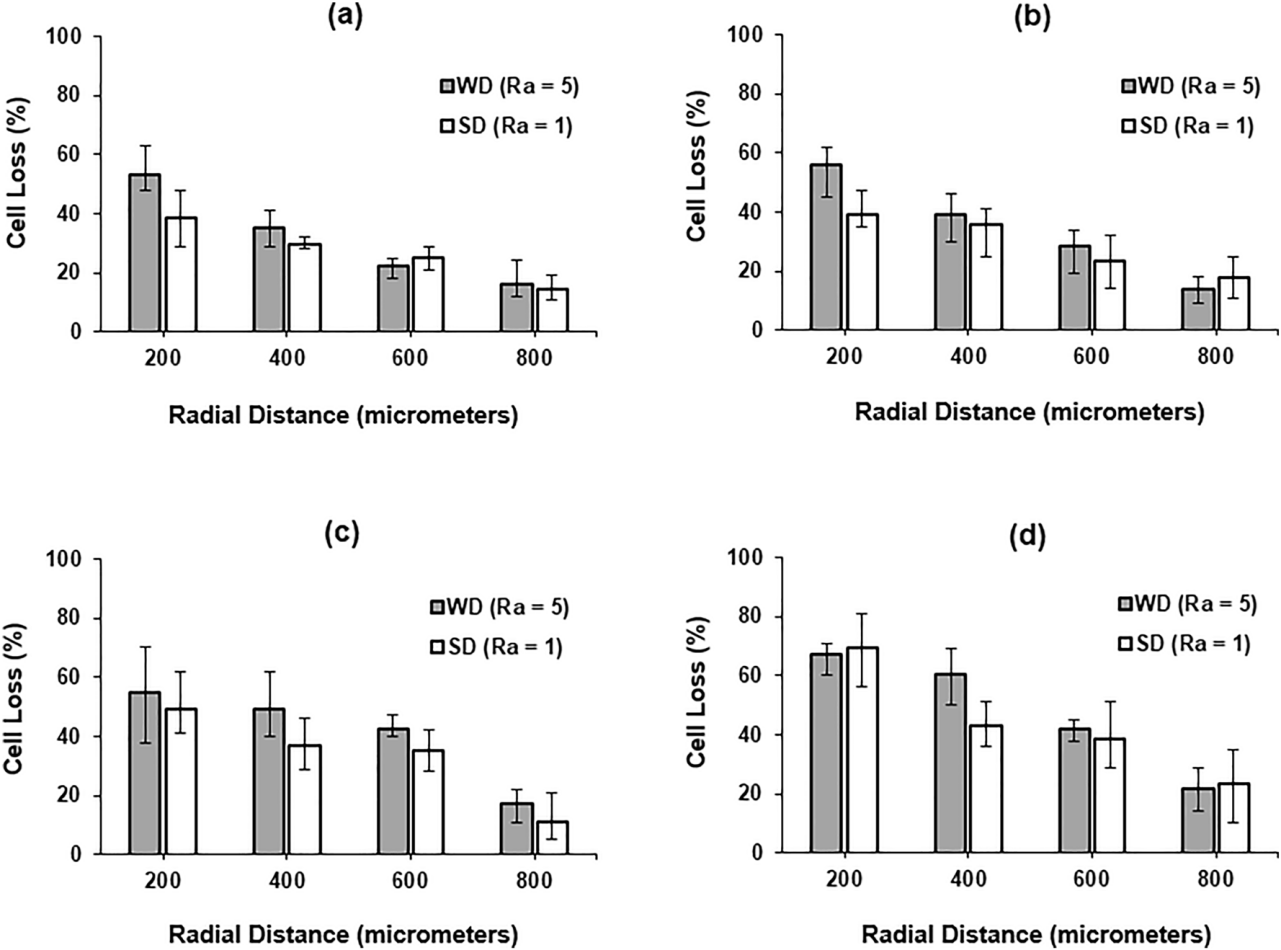
Cell loss at various depth measured along radial direction to hole wall (**a**) 4 mm; (**b**) 5 mm; (**c**) 6 mm; (**d**) 7 mm (drill speed—2000 rpm, WD—worn drill, SD—sharp drill).

In this section, some representative plots demonstrating the evolution of temperature, force and torque during bone drilling, bone histology and variation of the cell loss for two levels of drill-bit roughness are provided (Figs. 4, 5, 6, 7, 8, 9). A detailed statistical analysis of the cell damage caused by combination of drilling parameters is also presented. An area enclosed by a ring with a width of 200 µm in the radial direction (see Fig. 8) around the edge of the hole was selected to calculate the fraction of damage cell for the representative plots. The cell damage is the ratio of the number of empty lacunae without osteocytes to the total number of osteocytes in the selected region. Cell viability was measured at a depth of 200 μm from the cut surface of the hole since their activity at this depth surrounding the fixation devices was expected to be critical for osseointegration.

The number of disappeared osteocytes within the 200 µm distance from the edge of the hole was correlated with drill edge roughness, drill speed and drilling depth. The levels of bone temperature, axial force and torque were simultaneously measured at different depths of drilling along the cortex, together with the extent of biological damage (cell loss). The number of empty lacunae surrounding the cut surface was calculated along the radial direction for five levels of depths (3–7 mm). All the measured values of force, torque and temperature were recorded at their peaks during drilling. The effects of drill roughness and drilling depth on bone temperature, force, torque, and cell damage in combination with a range of drilling speeds were assessed. Each data point in the respective plots represents the average of three consecutive tests using a sharp (new) or worn drill and the same drilling parameters; the drill bits had the lowest and highest level of surface roughness—1 µm for a sharp drill bit and 5 µm for a worn one.

Temperature evolution in bone during drill penetration for two different depths and for sharp drill and worn drill bits is shown in Fig. 4. The measurement was recorded when the drill bits approached the thermocouple after few seconds following the start of the drilling process. The temperature was observed to increase sharply with time, attaining the peak value when the corresponding depth was reached. The maximum temperature recorded at a depth of 3 mm was 49 °C and 46 °C for a worn drill and a sharp drill, respectively. Similarly, at a depth of 7 mm, the maximum respective temperatures were 73 °C and 69 °C using a worn drill and a sharp drill. The bone temperature decreased slowly towards the room temperature when the drill was removed. For similar drilling conditions, the worn drill induced higher temperature in bone compared to the sharp drill.

The axial thrust force and torque measured for both types of drills are shown in Fig. 5. Similar to the bone temperature, the force was found to increase initially sharply with time, attaining a maximum peak value. The drilling force was found to have a somewhat higher value when the worn drill was used: its maximum was 57 N and 49 N for a worn drill a sharp drill, respectively. On the other hand, the torque for the worn drill increased from 4 to 14 N mm (250% increase) for the drilling depth changed form 3 mm to 7 mm. Similarly, an increase from 7 to 21 N mm was recorded for the same change in drilling depth in case of the sharp drill (Fig. 5). The data points for worn and sharp drills were fit using exponential and polynomial functions, respectively, in Fig. 5b. The torque was significantly lower for the worn drill compared to the sharp one for all drilling parameters and depths. There was also a significant reduction in torque when the drill speed was increased from 1000 to 3000 rpm.

A significant increase in bone temperature was noted for both types of drills when the drilling depth increased (Fig. 6). A temperature value of 68 °C was measured at 7 mm depth compared to 42 °C at 3 mm depth using the sharp drill. A similar trend in bone temperature was also noted for the worn drill. Increases by 48% and 21% in the force values were recorded when the drilling depth was changed from 3 to 7 mm for the sharp and the worn drills, respectively (Fig. 6).

Histology plots showing lacunae with osteocytes and empty lacunae, indicating disappeared osteocytes, are shown in Fig. 7. Histology results for compact bone specimens presenting osteons, Haversian canals, osteocytes, and lacunae are given in Fig. 8. Viable osteocytes, appeared as darker spots within the lacunae in histological images, are indicated by arrows, while locations of empty lacunae are encircled. Haversian canals, which allow blood vessels and nerves to grow through them, are enclosed with rectangles. Disappearances of the osteocytes from their chambers (lacunae) indicated the death of cells and were referred as “damage” in this study. Only some osteocytes and lacunae are highlighted to avoid the excessive use of labels. A comparison of the damaged cells resulting from drilling with the sharp and the worn drills at two different drilling depths with drill speed of 2000 rpm is provided in Fig. 8. A quantitative assessment indicated that more cells disappeared from lacunae when drilling was performed with the worn drill. Also, more cell damage was found at higher drilling depth.

The calculated levels of cell loss (damage) at four distances measured from the hole-wall in the radial direction and at different drilling depths for the sharp drill and the worn drill are shown in Fig. 9. The error bar of each data point denotes the upper and lower bounds of the measured values. The cell damage was found to grow significantly when the drilling depth increased. This may be attributed to the increased level of drilling force, torque and bone temperature produced at higher drilling depth. As expected, higher damage was observed for the worn drill (compared to the sharp drill) at all drilling depths. A decreasing pattern in damage with distance from the cut surface in the radial direction was established for all depths. The data obtained for the 3 mm drilling depth were used in the statistical analysis below.

Table B in Supplementary Data shows the grey relational coefficient related to the four responsible variables under consideration and the unified value of these response variables in the form of GRG. A higher value of GRG designates better results for a multi-response variable as it indicates that the experimental data are closer to the ideal normalized value. From Table B, it is evident that the highest grey relational grade was 0.898 related to the experimental set-up 26, where the levels of drill roughness, drill speed and drilling depth were fixed at 1 µm, 2000 rpm and 3 mm, respectively. The worst result was obtained for the experimental set-up 41, where the drill roughness, drill speed and drilling depth had the values of 4 µm, 2000 rpm and 7 mm, respectively.

### ANOVA analysis

Table A provided in Supplementary Data shows all the experimental setups and the observed data for the four response variables at these setups. In the table, the observed data are the average value for three consecutive drill tests, carried out at each set and the standard deviation. The average data were converted into GRG values using the GRA method as discussed in “Statistical Analysis” section. Analysis of variance (ANOVA) was implemented to check the effect of drill parameters on GRG. It was observed that the normal probability plot of GRG data had a near-linear behavior. Further, the pattern of residual plot was satisfactory as the residuals were contained in a horizontal band with random fluctuations inside it. This confirmed that the distribution of data was normal. This “normality” strengthened the findings of the variance and other statistical analysis. The result of ANOVA analysis is presented in Table 3. The obtained F- and P-values indicate that the GRG value was significantly affected by the drilling depth followed by drill roughness. The drill speed was found to have the least impact on the GRG value. Table 3 also provides the predicted regression equation for GRG versus drill roughness, drill speed and drilling depth. The absolute error between the actual GRG value and the value predicted based on the regression equation was found to vary between 0.033 and 16.4%. The average absolute error was found to be only 3.4%.

**Table 3:** ANOVA table for drill roughness, drill speed, and drilling depth.

Method					
Factor coding	(− 1, 0, + 1)				

Factor information					
Factor	Type	Levels	Values		
Drill roughness	Fixed	5	1, 2, 3, 4, 5		
Drill speed	Fixed	5	1000, 1500, 2000, 2500, 3000		
Drill depth	Fixed	5	3, 4, 5, 6, 7		

Analysis of variance					
Source	DF	Adj SS	Adj MS	F-value	P-value
Drill roughness	4	0.035878	0.008969	4.98	0.003
Drill speed	4	0.004574	0.001143	0.63	0.641
Drill depth	4	0.414524	0.103631	57.53	0.000
Error	32	0.057641	0.001801		
Total	44	0.513712			

Model summary					
S	R-sq	R-sq(adj)	R-sq(pred)		
0.0424414	88.78%	84.57%	77.81%		

Regression equation					
GRG = 1.0377 − 0.08842 drill depth − 0.000011 drill speed − 0.01696 drill roughness					

**Figure 10:**
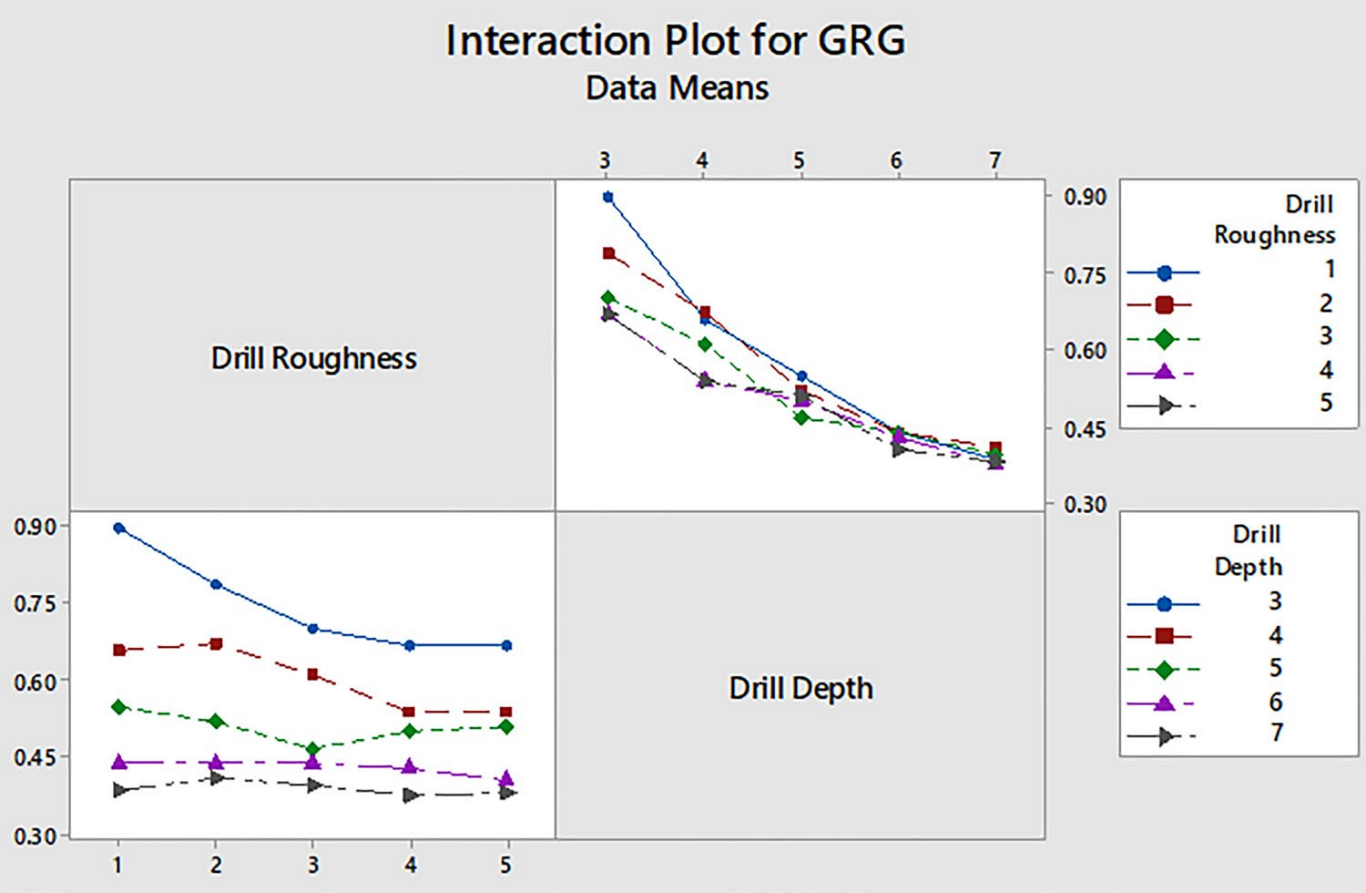
Interaction plots for GRG in terms of drill roughness and drilling depth.

Since the drill speed had the least impact on GRG, Fig. 10 shows only the interaction plot for GRG data with respect to drill roughness and drilling depth. From this plot it can be also inferred that the lower levels of drilling depth and the drill roughness are preferable for maximization of the GRG value. This is evident from the graphical results, and also scientifically more intuitive that the cell loss should be lower for smaller values of drilling depth and drill speed, and the use of the sharp drill (lower roughness or wear).

### Effect of drill roughness on force, temperature and cell loss

**Figure 11:**
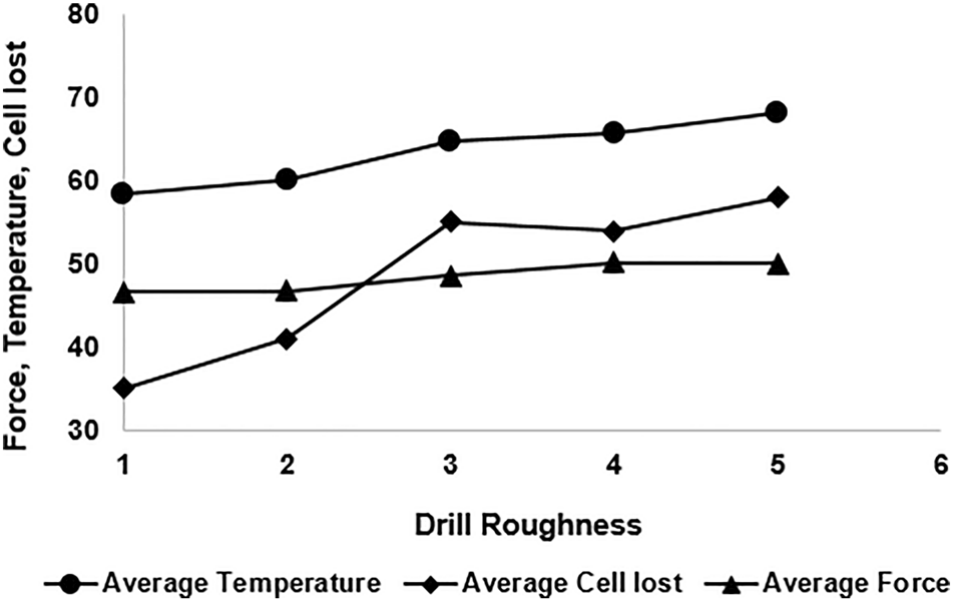
Effect of drill roughness on force, temperature and cell loss.

The relationships between drill roughness, force, temperature and cell loss are shown in Fig. 11. The obtained data demonstrate the average values over five trials conducted at each level of drill roughness. Apparently, with the increase in the drill roughness, all the responses, i.e., force, temperature and cell loss, increased. However, the rate, at which the force increased, was minimal. There was a rapid increase in temperature and cell loss when the drill roughness increased from 2 to 3 µm.

Further, an increasing trend of cell loss with the increase in the force and temperature was found. Obviously, the responses (force, temperature and cell loss) were impacted by the interaction of multiple process parameters that the research tried to optimize. However, it is clearly evident from the figure that with the increase in temperature and force, the cell loss increased; it was at maximum for the force and temperature at their highest values.

## Discussion

Surgical tools used for cutting bones do not have monitoring systems to evaluate biological damage, induced in the bone prior to fixation or repair. Currently, a surgical protocol does not provide surgeons or technicians with a clear indication on the number of holes a drill can produce before discarding it. Implementing such systems in clinics is extremely difficult due to the complex nature of drilling operations and real-time assessment of drill quality. In this study, the extent of bone damage was evaluated using the drilling parameters and conditions, similar to those in surgical clinics. A strong relationship between tool wear, temperature rise and cutting forces was confirmed in our study. Lower drill speeds produced lower levels of bone temperature and higher magnitudes of drilling force and torque. The increase in the drilling force using the lower drill speed may be attributed to inefficient evacuation of swarf from the drilling region. The lower levels of drilling force and torque may help to prevent overstressing the bone as well as to minimize the chances of drill breakage. The results obtained in this study, with regard to the effect of drill wear on bone temperature, were consistent with the published findings^43,44^. Still, relationships between the drilling force and the bone temperature reported in the literature complicated the identification of favorable drilling parameters with regard to the minimum damage in bone.

Elevated temperature in the bone at a cortex location of the drill-bit exit was due to the increased friction and trapping of heat in the hole. A decrease in bone temperature using lower drill speeds can be attributed to straining the bone material at lower rate and lower friction between the drill and the bone. Although the feed rate was kept constant in this study, it is known to significantly affect the drilling force, torque and bone temperature^2,6,20^. Microvibrations in the range up to 20 kHz imposed on the drill in the cutting direction can significantly reduce drilling force and the bone temperature^20^. The use of cutting tools with controlled micro-vibration (piezosurgery) demonstrated greater viability of cells and enhanced bone healing compared with a conventional cutting process^45–47^.

Osseointegration of mechanical fasteners attached to the bone is the prime indicator for the strength of fixation. The main factors contributing to biological damage in bone were the elevated temperature and overstressing the bone due to large drilling forces. The published research on bone drilling did not provide evidence of the contributions by the drilling force and temperature to biological damage in bone tissues. In addition, the studies investigating the effect of drill condition (sharp or blunt) on bone health are rare. Fewer necrotic cells, evidenced by empty lacunae, caused by drilling the bovine cortical bone, were found when the cutting force was increased^14^. The threshold level for temperature in drilling tibia bone was achieved when the drill penetrated to a depth of 50 mm^48^. An increase of osteocyte viability near the cutting region in sawing and burring of cortical bone was observed for cooling with saline solution^49^. Physiological saline solution routinely used in bone cutting procedures can reduce the drilling temperature and force. However, the contribution of cooling with saline solution to tissue damage is still unclear.

The cutting edges and the flank face of drills get blunted and the geometry of the cutting area distorts as the drill-bit use is increased. One of the focuses of this study was to investigate the effect of tool wear on bone health at drilling along the entire cortex of the bone. Generally, vibrational drilling may be helpful to avoid early blunting of the drill since the technique allows intermittent contact between the drill and bone. The results obtained in this study from the statistical analysis revealed minimum cell damage at shallow depths of drilling and lower drilling speeds and when using a sharper drill. However, an earlier study on ultrasonically assisted bone drilling^31^ recommended that detachment between the screw and bone (an undesirable occurrence) can be minimized for higher drill speeds. Similarly, a certain level of drilling depth is essential for correct insertion of the implant. Therefore, an arbitrary decision of lower drill speed and lowest drilling depth (for lower cell loss) is not always suitable, and surgeons can make an informed decision about the optimal combination of these three factors (drilling depth, drill roughness, and drill speed).

The intimate contact between the drilled bone and the implant is necessary for preventing early-stage failure of fixation. Quantitative analysis of the drilling-induced damage is vital for understanding and preventing mechanical failure of fixation devices anchoring bone. Nevertheless, this study is a step forward towards minimal invasive surgeries thanks to the obtained data on the damage in bone, using a series of experiments to study complex interaction of drilling parameters, drill condition and extent of damage induced in bone tissue. From the perspective of biology, the death of cells could significantly compromise the strength between implants and bone as well as remodeling process surrounding the drilled holes. Therefore, in-vivo studies should be conducted for the purpose of providing scientific data to the clinicians on preventing the onset of failure of fixation.

## Limitations of the current study and future research

1. Saline solution is routinely used in orthopedic surgical drilling to cool the cutting region and facilitate evacuation of tiny chips from the drilling region. In this study, cooling (irrigation), as used in real surgical drilling of bone, was not performed. It is expected that the acquired data for force, torque and temperature would vary if a coolant medium is used in the drilling experiments. Irrigation would have slightly decreased the temperature in the drilling region which could result in decrease in biological damage (cell loss) in the bone. In addition, Irrigation would reduce friction between the drill bit and the bone which could further decrease the drilling force and torque. The use of irrigation is strongly recommended in future studies pertinent to the biological damage in bone during drilling operation.
2. The temperature measurements were taken close to the cutting region, with an assumption that the temperature of the cut surface is the same as that of the cutting edges of the drill. However, this assumption may not be true in the case of trabecular (spongy) bone, where the effect of porosity cannot be ignored. A difference of 2 °C to 5 °C from the readings obtained from thermocouples and used in the analysis of biological damage in the current study was expected since the bead of the thermocouple was placed at a distance of approximately 1 mm from the drilling track. A small variation in the temperature data could also be possible due to inherent errors in the readings of the thermocouples as well as lower thermal conductivity of the bone material. This limitation could be addressed by using a non-contact method such as infrared thermography for temperature measurements in the drilling region. This would be possible if the correct value of thermal emissivity of the bone material is used in future studies.
3. Ultrasonic assisted drilling (UAD), where high-frequency vibration is superimposed on the drill movement in the cutting direction, is one of the novel technique reported in the literature on bone cutting. Unfortunately, this technique was not used in the current study since it would significantly increase variables or parameters involved in the statistical analysis. The use of micro-vibrations superimposed on the drill bit using controlled frequency would reduce temperature, force and torque. Further studies are suggested to investigate the relationship between vibration parameters such as frequency and amplitude and the extent of drill wear on the performance of bone drilling. The use of ultrasonic vibrations could alter the mechanics of the process and would change the results obtained on biological damage in bone. Further study is required to investigate the cell response of the bone to higher strain rates experienced in UAD. One of the interesting study would be the investigation on the effect of ultrasonic frequency on the extent of biological damage in bone in the presence of irrigation.
4. The choice of depth of 200 μm used in histology analysis was based on the assumption that the anchorage of bone with implant was more influenced at that depth. It would be interesting to find the relationship between the extent of biological damage and the radial distance from the cut surface. It is expected that the biological damage would exponential decrease if measured away from the cut surface in the radial direction. Further research is required to assess the biological damage at least up to 1000 μm away from the cut surface. It would be interesting to determine a distance from the cut surface where almost no biological damage would occur.
5. The roughness of the drill bit was measured along the cutting edges of the drill bits. Repeated use of drill bits would also decrease cutting performance of the chisel edge and alter roughness of the flanks. The obtained data on the temperature, force and torque would be slightly different if the surface profile of the chisel edge and flank were considered in calculating roughness of the drill bit. It is suggested to conduct further experiments by considering the aforementioned parameters in future investigations pertinent to the effect of surface roughness of the drill bit on the performance of drilling in bone.
6. An interesting topic for further research is the relationship between the extent of bone damage and osseointegration (bone formation) surrounding the implants and fixation elements. Obviously, the effect of drill wear on the performance of the process should be further investigated in clinical environment.

## Conclusions

Drilling experiments were performed on skeletally mature bovine bone using surgical drills with pre-defined levels of wear. The bone temperature was found to strongly depend on the drill wear, while an increase in the axial force was marginal when a blunt drill was used for drilling in bone. A worn drill could produce heat above a threshold level detrimental to bone cells at a depth equal to the wall thickness of an adult human bone. The torque in drilling was found to have a direct relationship with the depth of drilling; it decreased with increased drill wear. It is expected that surgeons and technicians have to apply more pressure on a worn drill when drilling through hard cortex of the bone, increasing the risk of drill breakage. Drill roughness has the highest impact on the safety of bone drilling in terms of biological damage. The most favorable drilling conditions were obtained with a sharper drill in combination with a medium drill speed and a lower drilling depth (if the latter is possible). A further study is required to investigate the cell response of the bone to variable frequency and measure the biological damage at a distance at least up to 1000 μm from the cut surface. The reported experiments were conducted using a medium sample size and only on the specimens excised from the middle part of the femur; so the study could be extended to different types of bones of the skeletal system.

## Supplementary Information

Supplementary Tables.

## Data Availability

The data generated or analyzed during this study are included in this published article (and its Supplementary Information files).
