# Supplementary material for: Effect of drill quality on biological damage in bone drilling
Source: Sci Rep. 2023 Apr 17;13:6234. doi: 10.1038/s41598-023-33381-y (PMC10110507; doi:10.1038/s41598-023-33381-y)
Supplement: Supplementary file 1 — Supplementary Tables. [file 41598_2023_33381_MOESM1_ESM.docx]

**Supplementary Data**

Table A. Experimental set-ups and observed data

| **Exp**  **No.** | **Experimental Set-up** | | | **Observed data for response variable** | | | | | | | |
| --- | --- | --- | --- | --- | --- | --- | --- | --- | --- | --- | --- |
|  | Drill Roughness  (µm) | Drill Speed  (rpm) | Drill Depth  (mm) | Force (N) | | Torque (N.m) | | Temp (oC) | | Cell lost (%) | |
|  |  |  |  | Avg | Stdev | Avg | Stdev | Avg | Stdev | Avg | Stdev |
| 1 | 1 | 1000 | 5 | 52 | 2.08 | 18 | 1.00 | 51 | 2.52 | 28 | 2.15 |
| 2 | 1 | 1500 | 5 | 47 | 1.73 | 16 | 1.15 | 52 | 1.53 | 30 | 1.53 |
| 3 | 1 | 2000 | 5 | 47 | 1.53 | 16 | 0.62 | 58 | 1.00 | 39 | 1.15 |
| 4 | 1 | 2500 | 5 | 43 | 1.15 | 14 | 1.56 | 64 | 1.53 | 40 | 1.53 |
| 5 | 1 | 3000 | 5 | 44 | 2.08 | 14 | 2.62 | 67 | 3.06 | 38 | 2.31 |
| 6 | 2 | 1000 | 5 | 51 | 1.00 | 18 | 1.00 | 52 | 2.31 | 25 | 1.73 |
| 7 | 2 | 1500 | 5 | 49 | 1.53 | 17 | 1.15 | 54 | 1.15 | 35 | 1.92 |
| 8 | 2 | 2000 | 5 | 48 | 2.00 | 16 | 1.15 | 61 | 1.53 | 50 | 0.78 |
| 9 | 2 | 2500 | 5 | 44 | 1.53 | 14 | 0.54 | 65 | 2.00 | 45 | 2.08 |
| 10 | 2 | 3000 | 5 | 42 | 2.00 | 13 | 1.25 | 68 | 1.73 | 49 | 1.53 |
| 11 | 3 | 1000 | 5 | 54 | 1.53 | 14 | 1.54 | 57 | 1.53 | 55 | 1.15 |
| 12 | 3 | 1500 | 5 | 51 | 1.00 | 12 | 0.75 | 63 | 2.52 | 50 | 1.00 |
| 13 | 3 | 2000 | 5 | 50 | 1.00 | 13 | 1.69 | 66 | 1.53 | 50 | 1.15 |
| 14 | 3 | 2500 | 5 | 45 | 2.00 | 12 | 1.15 | 66 | 4.05 | 59 | 2.00 |
| 15 | 3 | 3000 | 5 | 43 | 0.58 | 11 | 1.86 | 72 | 3.21 | 61 | 0.58 |
| 16 | 4 | 1000 | 5 | 56 | 1.00 | 13 | 2.14 | 55 | 0.58 | 50 | 1.00 |
| 17 | 4 | 1500 | 5 | 47 | 1.73 | 11 | 2.06 | 65 | 2.00 | 49 | 2.08 |
| 18 | 4 | 2000 | 5 | 56 | 3.04 | 6 | 0.69 | 65 | 1.00 | 51 | 0.00 |
| 19 | 4 | 2500 | 5 | 49 | 1.53 | 7 | 0.58 | 66 | 3.00 | 60 | 2.31 |
| 20 | 4 | 3000 | 5 | 43 | 0.58 | 7 | 2.15 | 78 | 1.00 | 60 | 1.00 |
| 21 | 5 | 1000 | 5 | 56 | 3.00 | 8 | 1.15 | 60 | 1.15 | 53 | 0.58 |
| 22 | 5 | 1500 | 5 | 48 | 1.00 | 8 | 1.65 | 68 | 1.15 | 55 | 1.53 |
| 23 | 5 | 2000 | 5 | 56 | 1.53 | 7 | 1.15 | 67 | 2.52 | 56 | 0.58 |
| 24 | 5 | 2500 | 5 | 48 | 2.31 | 6 | 0.00 | 71 | 2.08 | 65 | 1.15 |
| 25 | 5 | 3000 | 5 | 42 | 2.08 | 5 | 1.00 | 75 | 1.73 | 60 | 0.58 |
| 26 | 1 | 2000 | 3 | 33 | 0.69 | 7 | 1.15 | 46 | 1.00 | 25 | 2.64 |
| 27 | 1 | 2000 | 4 | 46 | 2.00 | 8 | 1.00 | 47 | 3.21 | 38 | 2.31 |
| 28 | 1 | 2000 | 6 | 48 | 1.00 | 19 | 0.58 | 65 | 1.73 | 49 | 1.53 |
| 29 | 1 | 2000 | 7 | 49 | 1.53 | 21 | 1.00 | 68 | 2.46 | 69 | 0.88 |
| 30 | 2 | 2000 | 3 | 41 | 0.58 | 7 | 1.15 | 43 | 3.58 | 31 | 0.00 |
| 31 | 2 | 2000 | 4 | 48 | 2.08 | 6 | 1.37 | 48 | 2.00 | 38 | 2.08 |
| 32 | 2 | 2000 | 6 | 49 | 0.78 | 17 | 0.89 | 68 | 1.73 | 48 | 1.53 |
| 33 | 2 | 2000 | 7 | 49 | 1.53 | 21 | 1.15 | 72 | 2.89 | 50 | 1.73 |
| 34 | 3 | 2000 | 3 | 40 | 2.08 | 5 | 0.58 | 50 | 1.53 | 45 | 2.16 |
| 35 | 3 | 2000 | 4 | 48 | 1.53 | 6 | 1.00 | 53 | 1.15 | 45 | 1.42 |
| 36 | 3 | 2000 | 6 | 52 | 2.31 | 13 | 1.00 | 71 | 1.73 | 50 | 2.08 |
| 37 | 3 | 2000 | 7 | 50 | 1.53 | 16 | 0.48 | 74 | 0.58 | 65 | 1.53 |
| 38 | 4 | 2000 | 3 | 45 | 0.00 | 5 | 1.25 | 51 | 1.73 | 42 | 0.58 |
| 39 | 4 | 2000 | 4 | 52 | 0.58 | 7 | 0.58 | 60 | 0.58 | 49 | 1.15 |
| 40 | 4 | 2000 | 6 | 54 | 1.15 | 14 | 1.00 | 70 | 0.58 | 50 | 2.31 |
| 41 | 4 | 2000 | 7 | 56 | 1.00 | 14 | 0.00 | 73 | 2.52 | 75 | 1.73 |
| 42 | 5 | 2000 | 3 | 47 | 1.73 | 4 | 0.00 | 50 | 2.00 | 50 | 0.58 |
| 43 | 5 | 2000 | 4 | 54 | 1.53 | 6 | 0.58 | 60 | 2.65 | 53 | 2.08 |
| 44 | 5 | 2000 | 6 | 57 | 1.00 | 14 | 0.00 | 73 | 1.53 | 55 | 1.53 |
| 45 | 5 | 2000 | 7 | 57 | 2.08 | 14 | 1.15 | 74 | 1.53 | 68 | 0.58 |

**Table B.** GRC and GRC values for observed data

| **Exp No** | **Grey relational coefficient** | | | | **GRG** | **Exp No** | **Grey relational coefficient** | | | | **GRG** |
| --- | --- | --- | --- | --- | --- | --- | --- | --- | --- | --- | --- |
|  | Force | Torque | Temp | Cell lost |  |  | Force | Torque | Temp | Cell lost |  |
| 1 | 0.387 | 0.378 | 0.686 | 0.893 | 0.586 | 24 | 0.444 | 0.810 | 0.385 | 0.385 | 0.506 |
| 2 | 0.462 | 0.415 | 0.660 | 0.833 | 0.592 | 25 | 0.571 | 0.895 | 0.354 | 0.417 | 0.559 |
| 3 | 0.462 | 0.415 | 0.538 | 0.641 | 0.514 | 26 | 1.000 | 0.739 | 0.854 | 1.000 | **0.898** |
| 4 | 0.545 | 0.459 | 0.455 | 0.625 | 0.521 | 27 | 0.480 | 0.680 | 0.814 | 0.658 | 0.658 |
| 5 | 0.522 | 0.459 | 0.422 | 0.658 | 0.515 | 28 | 0.444 | 0.362 | 0.443 | 0.510 | 0.440 |
| 6 | 0.400 | 0.378 | 0.660 | 1.000 | 0.610 | 29 | 0.429 | 0.333 | 0.412 | 0.362 | 0.384 |
| 7 | 0.429 | 0.395 | 0.614 | 0.714 | 0.538 | 30 | 0.600 | 0.739 | 1.000 | 0.806 | 0.786 |
| 8 | 0.444 | 0.415 | 0.493 | 0.500 | 0.463 | 31 | 0.444 | 0.810 | 0.778 | 0.658 | 0.672 |
| 9 | 0.522 | 0.459 | 0.443 | 0.556 | 0.495 | 32 | 0.429 | 0.395 | 0.412 | 0.521 | 0.439 |
| 10 | 0.571 | 0.486 | 0.412 | 0.510 | 0.495 | 33 | 0.429 | 0.333 | 0.376 | 0.500 | 0.410 |
| 11 | 0.364 | 0.459 | 0.556 | 0.455 | 0.458 | 34 | 0.632 | 0.895 | 0.714 | 0.556 | 0.699 |
| 12 | 0.400 | 0.515 | 0.467 | 0.500 | 0.470 | 35 | 0.444 | 0.810 | 0.636 | 0.556 | 0.611 |
| 13 | 0.414 | 0.486 | 0.432 | 0.500 | 0.458 | 36 | 0.387 | 0.486 | 0.385 | 0.500 | 0.439 |
| 14 | 0.500 | 0.515 | 0.432 | 0.424 | 0.468 | 37 | 0.414 | 0.415 | 0.361 | 0.385 | 0.393 |
| 15 | 0.545 | 0.548 | 0.376 | 0.410 | 0.470 | 38 | 0.500 | 0.895 | 0.686 | 0.595 | 0.669 |
| 16 | 0.343 | 0.486 | 0.593 | 0.500 | 0.480 | 39 | 0.387 | 0.739 | 0.507 | 0.510 | 0.536 |
| 17 | 0.462 | 0.548 | 0.443 | 0.510 | 0.491 | 40 | 0.364 | 0.459 | 0.393 | 0.500 | 0.429 |
| 18 | 0.343 | 0.810 | 0.443 | 0.490 | 0.521 | 41 | 0.343 | 0.459 | 0.368 | 0.333 | **0.376** |
| 19 | 0.429 | 0.739 | 0.432 | 0.417 | 0.504 | 42 | 0.462 | 1.000 | 0.714 | 0.500 | 0.669 |
| 20 | 0.545 | 0.739 | 0.333 | 0.417 | 0.509 | 43 | 0.364 | 0.810 | 0.507 | 0.472 | 0.538 |
| 21 | 0.343 | 0.680 | 0.507 | 0.472 | 0.500 | 44 | 0.333 | 0.459 | 0.368 | 0.455 | 0.404 |
| 22 | 0.444 | 0.680 | 0.412 | 0.455 | 0.498 | 45 | 0.333 | 0.459 | 0.361 | 0.368 | 0.380 |
| 23 | 0.343 | 0.739 | 0.422 | 0.446 | 0.488 |  |  |  |  |  |  |
